# Supplementary material for: Haplotype diversity of Plasmodium falciparum circumsporozoite protein prior to malaria vaccine implementation in Kaelé Health District, Cameroon, 2022–2023
Source: Front Parasitol. 2026 Apr 7;5:1749169. doi: 10.3389/fpara.2026.1749169 (PMC13097004; doi:10.3389/fpara.2026.1749169)

SUPPLEMENTARY MATERIALS

Haplotype Diversity of *Plasmodium falciparum* Circumsporozoite Protein Prior to Malaria Vaccine Implementation in Kaelé Health District, Cameroon, 2022-2023

Innocent M. Ali, Brenda Vasquez Martinez, Valery P. K. Tchuenkam, Jacob M. Sadler, Catherine C. Gorman, Sandrine E. Nsango, Voundi Voundi Junior, Ateba Joel, Wanda Grace, Jeffrey A. Bailey, Rhoel R. Dinglasan, Jessica T. Lin, Jonathan J. Juliano

Supplemental Material………… 2
Supplemental Table 1………… 3
Supplemental Table 2………… 5
Supplemental Table 3………… 6
Supplemental Table 4………… 8
Supplemental Table 5………… 11
Supplemental Table 6………… 14
Supplemental Table 7………… 16
Supplemental Table 8………… 19
Supplemental Figure 1………… 20
Supplemental Figure 2………… 21
Supplemental Figure 3………… 22
Supplemental Figure 4………… 23

Supplemental Material

Participant recruitment: Samples processed in this study represented positive samples from a household survey conducted after the implementation of the 2022 SMC survey in northern Cameroon. A household survey was conducted in Mapoussere to measure community parasite carriage 30-45 days after the SMC campaign. Villages/neighbourhoods in each health area were selected through a two-stage sampling process in the health area. In the first step, localities were selected using probability proportional to the size (Toure et al., 2024) based on the 2021 SMC household listing and micro-planning. Simple random sampling of neighbourhoods/villages was then used to identify households. In each household, an SMC-eligible child (3-59 months) was screened alongside a child 5-14 years old. In total, 900 children were screened in the health area. For each RDT-positive sample, light microscopy was performed to confirm infection status, and the sample was either used/not used for 4CAST depending on parasite status.

REFERENCES

Toure, M., Shaffer, J. G., Sanogo, D., Keita, S., Keita, M., Kane, F., et al. (2024). Seasonal Malaria Chemoprevention Therapy in Children Up To 9 Years of Age: Protocol for a Cluster-Randomized Trial Study. *JMIR Res Protoc* 13, e51660.

Supplemental Table 1. Control Sample Sequencing

Control	# reps	# with detected haplotype	Median # of detected haplotypes	Range	# Reps with Haplotype missed	Haplotype 1*		Haplotype 2*	
						Mean Freq.	Range	Mean Freq.	Range
csp									
10 p/µl	12	4	1	1-1	0	100%	100%-100%	-	-
100 p/µl	12	12	1	1-1	0	100%	100%-100%	-	-
1,000 p/µl	12	12	1	1-1	0	100%	100%-100%	-	-
Mix 1 (80:20)	10	10	2	1-2	2	92.3%	86.6%-100%	10.9%	7.4%-13.4%
Mix 2 (95:5)	10	10	2	1-2	5	97.5%	94.4%-100%	5.0%	4.5%-5.6%
ama1									
10 p/µl	12	7	1	1-1	0	100%	100%-100%	-	-
100 p/µl	12	12	1	1-1	0	100%	100%-100%	-	-
1,000 p/µl	12	12	1	1-1	0	100%	100%-100%	-	-
Mix 1 (80:20)	10	10	2	2-2	0	77.6%	73.5%-82.5%	22.7%	17.5%-26.5%
Mix 2 (95:5)	10	10	2	1-2	2	97.0%	95.3%-98.7%	3.8%	2.6%-4.9%
sera2									
10 p/µl	12	6	1	1-1	0	100%	100%-100%	-	-
100 p/µl	12	12	1	1-1	0	100%	100%-100%	-	-
1,000 p/µl	12	12	1	1-1	0	100%	100%-100%	-	-
Mix 1 (80:20)	10	10	2	2-2	0	79.2%	76.7%-81.5%	20.8%	18.5%-23.9%
Mix 2 (95:5)	10	10	2	2-2	0	93.5%	93.1%-94.7%	6.5%	5.3%-6.9%
trap									
10 p/µl	12	0	-	-	-	-	-	-	-
100 p/µl	12	12	1	1-1	0	100%	100%-100%	-	-
1,000 p/µl	12	12	1	1-1	0	100%	100%-100%	-	-
Mix 1 (80:20)	10	9	2	1-3	1[#]	82.7%	76.3%-83.0%	16.8%	12.4%-19.3%
Mix 2 (95:5)	10	10	1	1-1	10	100%	100%-100%	-	-

*: Of replicates with detectable haplotypes (zeros not counted).

#: Two samples with a false minor variant. Both were the same haplotype, at 10.8% and 10.3% frequency, within controls. The haplotype contained 4 mutations; the first 2 were 3D7-like, the second 2 were 7G8-like, suggesting this may be a chimera. This haplotype was not found in any samples.

Supplemental Table 2. Individual Level Genotyping Data.

Please see the uploaded Excel sheet.

Supplemental Table 3. *csp* Haplotypes Detected

Haplotype	# Samples Detected	Sequence
PfCSP.00	9	TATTAATCCTATTGAACTATTTACGACATTAAACACACTGGAACATTTTTCCATTTTACAAATTTTTTTTTCAATATCATTTTCATAATCTAATTGGTCTTTAGG<br>TTTATTAGCAGAGCCAGGCTTTATTCTAACTTGAATACCATTTCCACAAGTTACACTACATGGGGACCATTCAGTTGAAAGAGAATTTTGTATTGTCTTTAA<br>ATATTGTTCTATGTGCTTATCACTTGGTTCTTCGTTATTATTATTTTTTACAGCATTGTTGGCTTTAGCATTTTCATCT
PfCSP.01	7	TATTAATCCTATTGAACTATTTACGACATTAAACACACTGGAACATTTTTCCATTTTACAAATTTTTTTTTCAATATCATTTGCATAATCTAATTCGTCTTTAGG<br>TTTATTAGCAGAGCCAGGCTTTATTCTAACTTGAATACCATTTCCACAAGTTACACTACATGGGGACCATTCAGTTGAAAGAGAATTTTGTATTTTGTTTAA<br>ATATTCTTTTATGTGCTTATCACTTGGTTCTTCGTTATTATTATTTTTTACAGCACTGTTGGCATTAGCATTTTCATCT
PfCSP.02	4	TATTAATCCTATTGAACTATTTACGACATTAAACACACTGGAACATTTTTCCATTTTACAAATTTTTTTTTCAATATCATTTGCATAATCTAATTGGTCTTTAGG<br>TTTATTAGCAGAGCCAGGCTTTATTCTAACTTGAATACCATTTCCACAAGTTACACTACATGGGGACCATTCAGTTGAAAGAGAATTTTTTATTTTGTTTAA<br>ATATTTTTCTATGTGCTGATCACTTGGTTCTTCGTTATTATTATTTTTTACAGCATTGTTGGCATTAGCATTTTCATCT
PfCSP.03	3	TATTAATCCTATTGAACTATTTACGACATTAAACACACTGGAACATTTTTCCATTTTACAAATTTTTTTTTCAATATCATTTGCATAATCTAATTGGTCTTTAGG<br>TTTATTAGCAGAGCCAGGCTTTATTCTAACTTGAATACCATTTCCACAAGTTACACTACATGGGGACCATTCAGTTGAAAGAGAATTTTGTATTGTCTTTAA<br>ATATTTTTCTATGTGCTGATCACTTGGTTCTTCGTTATTATTATTTTTTACAGCATTGTTGGCATTAGCATTTTCATCT
PfCSP.04	3	TATTAATCCTATTGAACTATTTACGACATTAAACACACTGGAACATTTTTCCATTTTACAAATTTTTTTTTCAATATCATTTGCATAATCTAATTGGTCTTTAGG<br>TTTATTAGCAGAGCCAGGCTTTATTCTAACTTGAATACCATTTCCACAAGTTACACTACATGGGGACCATTCAGTTGAAAGAGAATTTTTTATTGTCTTTAA<br>ATATTTTTCTATGTGCTGATCACTTGGTTCTTCGTTATTATTATTTTTTACAGCATTGTTGGCATTAGCATTTTCATCT
PfCSP.05	2	TATTAATCCTATTGAACTATTTACGACATTAAACACACTGGAACATTTTTCCATTTTACAAATTTTTTTTTCAATATCATTTTCATAATCTAATTCGTCTTTAGA<br>TTTACCAGCAGAGCCAGGCTTTATTCTAACTTGAATACCATTTCCACAAGTTACACTACATGGGGACCATTCAGTTGAAAGAGAATTTTGTATTTTCTTTA<br>AATATTGTTCTATGTGCTTATCACTTGGTTCTTCGTTATTATTATTTTTTACAGCATTGTTGGCATTAGCATTTTCATCT
PfCSP.06	2	TATTAATCCTATTGAACTATTTACGACATTAAACACACTGGAACATTTTTCCATTTTACAAATTTTTTTTTCAATATCATTTGCATAATCTAATTGGTCTTTAGG<br>TTTATTAGCAGAGCCAGGCTTTATTCTAACTTGAATACCATTTCCACAAGTTACACTACATGGGGACCATTCAGTTGAAAGAGAATTTTTTATTATCTTTAA<br>ATATTTTTCTATGTGCTGATCACTTGGTTCTTCGTTATTATTATTTTTTACAGCATTGTTGGCATTAGCATTTTCATCT
PfCSP.07	1	TATTAATCCTATTGAACTATTTACGACATTAAACACACTGGAACATTTTTCCATTTTACAAATTTTTTTTTCAATATCATTTTCATAATTTAATTCGTCTTTAGG<br>TTTATTAGCAGAGCCAGGCTTTATTCTAACTTGAATACCATTTCCACAAGTTACACTACATGGGGACCATTCAGTTGAAATAGAATTTTTTATTTTGTTTAA<br>ATATTGTTCTATGTGCTTATCACTTGGTTCTTCGTTATTATTATTTTTTACAGCATTGTTGGCATTAGCATTTTCATCT
PfCSP.08	1	TATTAATCCTATTGAACTATTTACGACATTAAACACACTGGAACATTTTTCCATTTTACAAATTTTTTTTTCAATATCATTTTCATAATTTAATTCCTCTTTAGG<br>TTTATTAGCAGAGCCAGGCTTTATTCTAACTTGAATACCATTTCCACAAGTTACACTACATGGGGACCATTCAGTTGAAAGAGAATTTTGTATTCTCTTTAA<br>ATATTCTGTTATGTGCTTATCACTTGGTTCTTCGTTATTATTATTTTTTACAGCATTGTTGGAATTAGCATTTTCATCT
PfCSP.09	1	TATTAATCCTATTGAACTATTTACGACATTAAACACACTGGAACATTTTTCCATTTTACAAATTTTTTTTTCAATATCATTTTCATAATCTAATTCGTTTTTAGAT<br>TTACCAGCAGAGCCAGGCTTTATTCTAACTTGAATACCATTTCCACAAGTTACACTACATGGGGACCATTCAGTTGAAAGAGAATTTTGTATTCTCTTTAA<br>ATATTCTGTTATGTGCTTATCACTTGGTTCTTCGTTATTATTATTTTTTACAGCATTGTTGCCATTAGCATTTTCATCT
PfCSP.10	1	TATTAATCCTATTGAACTATTTACGACATTAAACACACTGGAACATTTTTCCATTTTACAAATTTTTTTTTCAATATCATTTTCATAATCTAATTGGTCTTTAGG<br>TTTATTAGCAGAGCCAGGCTTTATTCTAACTTGAATACCATTTCCACAAGTTACACTACATGGGGACCATTCAGTTGAAAGAGAATTTTTTATTGTCTTTAA

		ATATTGTTCTATGTGCTTATCACTTGGTTCTTCGTTATTATTATTTTTTACAGCATTGTTGGCTTTAGCATTTTCATCT
PfCSP.11	1	TATTAATCCTATTGAACTATTTACGACATTAAACACACTGGAACATTTTTCCATTTTACAAATTTTTTTTTCAATATCATTTGCATAATCTAATTGGTCTTTAGGTTTATCAGCAGAGCCAGGCTTTATTCTAACTTGAATACCATTTCCACAAGTTACACTACATGGGGACCATTCAGTTGAAAGAGAATTTTGTATTCTCTTTAAATATTTTTCTATGTGCTGATCACTTGGTTCTTCGTTATTATTATTTTTTACAGCATTGTTGGCATTAGCATTTTCATCT
PfCSP.12	1	TATTAATCCTATTGAACTATTTACGACATTAAACACACTGGAACATTTTTCCATTTTACAAATTTTTTTTTCAATATCATTTGCATAATCTAATTGGTCTTTAGGTTTATTAGCAGAGCCAGGCTTTATTCTAACTTGAATACCATTTCCACAAGTTACACTACATGGGGACCATTCAGTTGAAAGAGAATTTTGTATTCTCTTTAAATATTTTTCTATGTGCTGATCACTTGGTTCTTCGTTATTATTATTTTTTACAGCATTGTTGGCATTAGCATTTTCATCT
PfCSP.13	1	TATTAATCCTATTGAACTATTTACGACATTAAACACACTGGAACATTTTTCCATTTTACAAATTTTTTTTTCAATATCATTTTCATAATTTAATTCGTCTTTAGGTTTATTAGCAGAGCCAGGCTTTATTCTAACTTGAATACCATTTCCACAAGTTACACTACATGGGGACCATTCAGTTGAAATAGAATTTTTTATTTTCTTTAAATATTCTTCTATGTGCTTATCACTTGGTTCTTCGTTATTATTATTTTTTACAGCATTGTTGGCATTAGCATTTTCATCT
PfCSP.14	1	TATTAATCCTATTGAACTATTTACGACATTAAACACACTGGAACATTTTTCCATTTTACAAATTTTTTTTTCAATATCATTTGCATAATCTAATTGGTCTTTAGGTTTATCAGCAGAGCCAGGCTTTATTCTAACTTGAATACCATTTCCACAAGTTACACTACATGGGGACCATTCAGTTGAAAGAGAATTTTGTATTTTCTTTAAATATTTTTCTATGTGCTGATCACTTGGTTCTTCGTTATTATTATTTTTTACAGCATTGTTGCCATTAGCATTTTCATCT
PfCSP.15	1	TATTAATCCTATTGAACTATTTACGACATTAAACACACTGGAACATTTTTCCATTTTACAAATTTTTTTTTCAATATCATTTTCATAATTTAATTCGTCTTTAGGTTTACCAGCAGAGCCAGGCTTTATTCTAACTTGAATACCATTTCCACAAGTTACACTACATGGGGACCATTCAGTTGAAATAGAATTTTGTATTCTCTTTAAATATTCTTTTATGTGCTTATCACTTGGTTCTTCGTTATTATTATTTTTTACAGCATTGTTGGCATTAGCATTTTCATCT
PfCSP.16	1	TATTAATCCTATTGAACTATTTACGACATTAAACACACTGGAACATTTTTCCATTTTACAAATTTTTTTTTCAATATCATTTTCATAATCTAATTCGTCTTTAGGTTTATTAGCAGAGCCAGGCTTTATTCTAACTTGAATACCATTTCCACAAGTTACACTACATGGGGACCATTCAGTTGAAAGAGAATTTTGTATTGTCTTTAAATATTGTTCTATGTGCTTATCACTTGGTTCTTCGTTATTATTATTTTTTACAGCATTGTTGGCTTTAGCATTTTCATCT
PfCSP.17	1	TATTAATCCTATTGAACTATTTACGACATTAAACACACTGGAACATTTTTCCATTTTACAAATTTTTTTTTCAATATCATTTGCATAATCTAATTGGTCTTTAGGTTTATTAGCAGAGCCAGGCTTTATTCTAACTTGAATACCATTTCCACAAGTTACACTACATGGGGACCATTCAGTTGAAAGAGAATTTTGTATTGTCTTTAAATATTTTTCTATGTGCTGATCACTTGGTTCTTCGTTATTATTATTTTTTACAGCATTGTTGGCTTTAGCATTTTCATCT
PfCSP.18	1	TATTAATCCTATTGAACTATTTACGACATTAAACACACTGGAACATTTTTCCATTTTACAAATTTTTTTTTCAATATCATTTTCATAATTTAATTCGTCTTTAGGTTTATTAGCAGAGCCAGGCTTTATTCTAACTTGAATACCATTTCCACAAGTTACACTACATGGGGACCATTCAGTTGAAAGAGAATTTTGTATTCTCTTTAAATATTTTTCTATGTGCTGATCACTTGGTTCTTCGTTATTATTATTTTTTACAGCATTGTTGGCATTAGCATTTTCATCT
PfCSP.19	1	TATTAATCCTATTGAACTATTTACGACATTAAACACACTGGAACATTTTTCCATTTTACAAATTTTTTTTTCAATATCATTTTCATAATCTAATTGGTCTTTAGGTTTATTAGCAGAGCCAGGCTTTATTCTAACTTGAATACCATTTCCACAAGTTACACTACATGGGGACCATTCAGTTGAAAGAGAATTTTTTATTGTCTTTAAATATTGTTCTATGTGCTTATCACTTGGTTCTTCGTTATTATTATTTTTTACAGCATTGTTGGCATTAGCATTTTCATCT
PfCSP.20	1	TATTAATCCTATTGAACTATTTACGACATTAAACACACTGGAACATTTTTCCATTTTACAAATTTTTTTTTCAATATCATTTTCATAATCTAATTCGTTTTTAGGTTTATCAGCAGAGCCAGGCTTTATTCTAACTTGAATACCATTTCCACAAGTTACACTACATGGGGACCATTCAGTTGAAAGAGAATTTTGTATTTTCTTTATATATTTTTCTATGTGCTGATCACTTGGTTCTTCGTTATTATTATTTTTTACAGCATTGTTGGCATTAGCATTTGCATCT
PfCSP.21	1	TATTAATCCTATTGAACTATTTACGACATTAAACACACTGGAACATTTTTCCATTTTACAAATTTTTTTTTCAATATCATTTGCATAATCTAATTGGTCTTTAGGTTTATTAGCAGAGCCAGGCTTTATTCTAACTTGAATACCATTTCCACAAGTTACACTACATGGGGACCATTCAGTTGAAATAGAATTTTGTATTTTGTTTAAATATTCTTTTATGTGCTTATCACTTGGTTCTTCGTTATTATTATTTTTTACAGCACTGTTGGCATTAGCATTTTCATCT

Supplemental Table 4. *ama1* Haplotypes Detected

Haplotype	# Samples Detected	Sequence
PfAMA1.00	23	TTTGGTAAAGGTATAATTATTGAGAATTCAAATACTACTTTTTTAAAACCGGTAGCTACGGGAAATCAAGATTTAAAAGATGGAGGTTTTGCTTTTCCTCC<br>AACAGAACCTCTTATATCACCAATGACATTAAATGGTATGAGAGATTTTTATAAAAATAATGAATATGTAAAAAATTTAGATGAATTGACTTT
PfAMA1.01	18	TTTGGTAAAGGTATAATTATTGAGAATTCAAATACTACTTTTTTAACACCGGTAGCTACGGGAAAACAAGATTTAAAAGATGGAGGTTTTGCTTTTCCTC<br>CAACAAATCCTCTTATATCACCAATGACATTAAATGGTATGAAAGATTTTTATAAAGATAATGAAGATGTAAAAAATTTAGATGAATTGACTTT
PfAMA1.02	17	TTTGGTAAAGGTATAATTATTGAGAATTCAAAAACTACTTTTTTAACACCGGTAGCTACGGAAAATCAAGATTTAAAAGATGGAGGTTTTGCTTTTCCTC<br>CAACAAATCCTCCTATGTCACCAATGACATTAAATGGTATGAGAGATTTATATAAAAATAATGAATATGTAAAAAATTTAGATGAATTGACTTT
PfAMA1.03	16	TTTGGTAAAGGTATAATTATTGAGAATTCAAATACTACTTTTTTAACACCGGTAGCTACGGGAAATCAAGATTTAAAAGATGGAGGTTTTGCTTTTCCTCC<br>AACAAAACCTCTTATGTCACCAATGACATTAGATGATATGAGACTTTTGTATAAAGATAATGAAGATGTAAAAAATTTAGATGAATTGACTTT
PfAMA1.04	15	TTTGGTAAAGGTATAATTATTGAGAATTCAAATACTACTTTTTTAACACCGGTAGCTACGGAAAATCAAGATTTAAAAGATGGAGGTTTTGCTTTTCCTCC<br>AACAAAACCTCTTATGTCACCAATGACATTAGATCAAATGAGACATTTTTATAAAGATAATAAATATGTAAAAAATTTAGATGAATTGACTTT
PfAMA1.05	12	TTTGGTAAAGGTATAATTATTGAGAATTCAAATACTACTTTTTTAACACCGGTAGCTACGGGAAATCAATATTTAAAAGATGGAGGTTTTGCTTTTCCTCC<br>AACAGAACCTCTTATGTCACCAATGACATTAGATGAAATGAGACATTTTTATAAAGATAATAAATATGTAAAAAATTTAGATGAATTGACTTT
PfAMA1.06	11	TTTGGTAAAGGTATAATTATTGAGAATTCAAATACTACTTTTTTAACACCGGTAGCTACGGAAAATCAAGATTTAAAAGATGGAGGTTTTGCTTTTCCTCC<br>AACAAATCCTCTTATATCACCAATGACATTAGATCATATGAGAGATTCTTATAAAAATAATGAATATGTAAAAAATTTAGATGAATTGACTTT
PfAMA1.07	11	TTTGGTAAAGGTATAATTATTGAGAATTCAAATACTACTTTTTTAACACCGGTAGCTACGGAAAATCAAGATTTAAAAGATGGAGGTTTTGCTTTTCCTCC<br>AACAGAACCTCTTATGTCACCAATGACATTAGATCAAATGAGACATTTTTATAAAGATAATAAATATGTAAAAAATTTAGATGAATTGACTTT
PfAMA1.08	11	TTTGGTAAAGGTATAATTATTGAGAATTCAAAAACTACTTTTTTAACACCGGTAGCTACGGAAAATCAAGATTTAAAAGATGGAGGTTTTGCTTTTCCTC<br>CAACAGAACCTCTTATGTCACCAATGACATTAGATGATATGAGACGTTTTTATAAAGATAATGAATATGTAAAAAATTTAGATGAATTGACTTT
PfAMA1.09	10	TTTGGTAAAGGTATAATTATTGAGAATTCAAATACTACTTTTTTAACACCGGTAGCTACGGAAAATCAAGATTTAAAAGATGGAGGTTTTGCTTTTCCTCC<br>AACAAAACCTCTTATATCACCAATGACATTAGATCAAATGAGAGATTTATATAAAAATAATGAATATGTAAAAAATTTAGATGAATTGACTTT
PfAMA1.10	9	TTTGGTAAAGGTATAATTATTGAGAATTCAAATACTACTTTTTTAACACCGGTAGCTACGGAAAATCAAGATTTAAAAGATGGAGGTTTTGCTTTTCCTCC<br>AACAAAACCTCTTATGTCACCAATGACATTAGATCAAATGAGAGATTTTTATAAAAATAATGAATATGTAAAAAATTTAGATGAATTGACTTT
PfAMA1.11	9	TTTGGTAAAGGTATAATTATTGAGAATTCAAAAACTACTTTTTTAACACCGGTAGCTACGGAAAATCAAGATTTAAAAGATGGAGGTTTTGCTTTTCCTC<br>CAACAAAACCTCTTATGTCACCAATGACATTAGATGATATGAGACTTTTGTATAAAGATAATGAAGATGTAAAAAATTTAGATGAATTGACTTT
PfAMA1.12	6	TTTGGTAAAGGTATAATTATTGAGAATTCAAATACTACTTTTTTAACACCGGTAGCTACGGAAAATCAAGATTTAAAAGATGGAGGTTTTGCTTTTCCTCC<br>AACAAATCCTCCTATGTCACCAATGACATTAGATCAAATGAGACATTTTTATAAAGATAATAAATATGTAAAAAATTTAGATGAATTGACTTT
PfAMA1.13	6	TTTGGTAAAGGTATAATTATTGAGAATTCAAATACTACTTTTTTAACACCGGTAGCTACGGAAAATCAAGATTTAAAAGATGGAGGTTTTGCTTTTCCTCC<br>AACAAATCCTCTTATATCACCAATGACATTAAATGGTATGAAAGATTTTTATAAAGATAATGAAGATGTAAAAAATTTAGATGAATTGACTTT
PfAMA1.14	6	TTTGGTAAAGGTATAATTATTGAGAATTCAAATACTACTTTTTTAAAACCGGTAGCTACGGGAAATCAAGATTTAAAAGATGGAGGTTTTGCTTTTCCTCC<br>AACAAATCCTCTTATATCACCAATGACATTAGATCAAATGAGACATTTTTATAAAGATAATGAAGATGTAAAAAATTTAGATGAATTGACTTT

PfAMA1.15	5	TTTGGTAAAGGTATAATTATTGAGAATTCAAATACTACTTTTTTAACACCGGTAGCTACGGGAAAACAAGATTTAAAAGATGGAGGTTTTGCTTTTCCTCCAACAAATCCTCTTATATCACCAATGACATTAGATCATATGAGAGATTTTTATAAAAAAAATGAATATGTAAAAAATTTAGATGAATTGACTTT
PfAMA1.16	5	TTTGGTAAAGGTATAATTATTGAGAATTCAAATACTACTTTTTTAACACCGGTAGCTACGGAAAATCAAGATTTAAAAGATGGAGGTTTTGCTTTTCCTCCAACAAAACCTCTTATGTCACCAATGACATTAGATGAAATGAGACATTTTTATAAAGATAATAAATATGTAAAAAATTTAGATGAATTGACTTT
PfAMA1.17	5	TTTGGTAAAGGTATAATTATTGAGAATTCAAAAACTACTTTTTTAACACCGGTAGCTACGGAAAATCAAGATTTAAAAGATGGAGGTTTTGCTTTTCCTCCAACAAATCCTCCTATGTCACCAATGACATTAGATGATATGAGACTTTTGTATAAAGATAATGAAGATGTAAAAAATTTAGATGAATTGACTTT
PfAMA1.18	4	TTTGGTAAAGGTATAATTATTGAGAATTCAAATACTACTTTTTTAAAACCGGTAGCTACGGAAAATCAAGATTTAAAAGATGGAGGTTTTGCTTTTCCTCCAACAAATCCTCTTATGTCACCAATGACATTAGATCATATGAGACATCTTTATAAAGATAATGAATATGTAAAAAATTTAGATGAATTGACTTT
PfAMA1.19	4	TTTGGTAAAGGTATAATTATTGAGAATTCAAATACTACTTTTTTAAAACCGGTAGCTACGGGAAATCAAGATTTAAAAGATGGAGGTTTTGCTTTTCCTCCAACAGAACCTCTTATATCACCAATGACATTAGATGATATGAGAGATTTTTATAAAAATAATGAATATGTAAAAAATTTAGATGAATTGACTTT
PfAMA1.20	4	TTTGGTAAAGGTATAATTATTGAGAATTCAAATACTACTTTTTTAACACCGGTAGCTACGGAAAATCAAGATTTAAAAGATGGAGGTTTTGCTTTTCCTCCAACAGAACCTCTTATGTCACCAATGACATTAGATCGTATGAGAGATTTTTATAAAAATAATGAAGATGTAAAAAATTTAGATGAATTGACTTT
PfAMA1.21	4	TTTGGTAAAGGTATAATTATTGAGAATTCAAATACTACTTTTTTAACACCGGTAGCTACGGGAAATCAATATTTAAAAGATGGAGGTTTTGCTTTTCCTCCAACAGAACCTCATATGTCACCAATGACATTAGATGAAATGAGACATTTTTATAAAGATAATAAATATGTAAAAAATTTAGATGAATTGACTTT
PfAMA1.22	3	TTTGGTAAAGGTATAATTATTGAGAATTCAAATACTACTTTTTTAACACCGGTAGCTACGGGAAAACAAGATTTAAAAGATGGAGGTTTTGCTTTTCCTCCAACAAATCCTCTTATATCACCAATGACATTAAATGGTATGAGAGATTTATATAAAAATAATGAAGATGTAAAAAATTTAGATGAATTGACTTT
PfAMA1.23	3	TTTGGTAAAGGTATAATTATTGAGAATTCAAATACTACTTTTTTAAAACCGGTAGCTACGGGAAATCAAGATTTAAAAGATGGAGGTTTTGCTTTTCCTCCAACAAATCCTCTTATATCACCAATGACATTAGATCATATGAGAGATTTTTATAAAAATAATGAATATGTAAAAAATTTAGATGAATTGACTTT
PfAMA1.24	3	TTTGGTAAAGGTATAATTATTGAGAATTCAAATACTACTTTTTTAAAACCGGTAGCTACGGGAAATCAAGATTTAAAAGATGGAGGTTTTGCTTTTCCTCCAACAGAACCTCTTATATCACCAATGACATTAAAGGGTATGAGAGATTTTTATAAAAATAATGAATATGTAAAAAATTTAGATGAATTGACTTT
PfAMA1.25	2	TTTGGTAAAGGTATAATTATTGAGAATTCAAATACTACTTTTTTAACACCGGTAGCTACGGAAAATCAAGATTTAAAAGATGGAGGTTTTGCTTTTCCTCCAACAAAACCTCTTATGTCACCAATGACATTAGATGATATGAGACTTTTGTATAAAGATAATGAAGATGTAAAAAATTTAGATGAATTGACTTT
PfAMA1.26	2	TTTGGTAAAGGTATAATTATTGAGAATTCAAATACTACTTTTTTAACACCGGTAGCTACGGAAAATCAAGATTTAAAAGATGGAGGTTTTGCTTTTCCTCCAACAAAACCTCATATGTCACCAATGACATTAGATGATATGAGACTTTTGTATAAAGATAATGAAGATGTAAAAAATTTAGATGAATTGACTTT
PfAMA1.27	2	TTTGGTAAAGGTATAATTATTGAGAATTCAAATACTACTTTTTTAAAACCGGTAGCTACGGGAAATCAAGATTTAAAAGATGGAGGTTTTGCTTTTCCTCCAACAAATCCTCTTATATCACCAATGACATTAAATGGTATGAAAGATTTTTATAAAGATAATGAAGATGTAAAAAATTTAGATGAATTGACTTT
PfAMA1.28	2	TTTGGTAAAGGTATAATTATTGAGAATTCAAAAACTACTTTTTTAACACCGGTAGCTACGGAAAATCAAGATTTAAAAGATGGAGGTTTTGCTTTTCCTCCAACAAAACCTCTTATGTCACCAATGACATTAGATGATATGAGAGATTTTTATAAAGATAATGAATATGTAAAAAATTTAGATGAATTGACTTT
PfAMA1.29	2	TTTGGTAAAGGTATAATTATTGAGAATTCAAATACTACTTTTTTAAAACCGGTAGCTACGGGAAATCAAGATTTAAAAGATGGAGGTTTTGCTTTTCCTCCAACAAATCCTCTTATATCACCAATGACATTAAATGGTATGAGAGATTTATATAAAAATAATGAAGATGTAAAAAATTTAGATGAATTGACTTT
PfAMA1.30	2	TTTGGTAAAGGTATAATTATTGAGAATTCAAAAACTACTTTTTTAACACCGGTAGCTACGGAAAATCAAGATTTAAAAGATGGAGGTTTTGCTTTTCCTCCAACAGAACCTCCTATGTCACCAATGACATTAGATGAAATGAGACATTTTTATAAAGATAATAAATATGTAAAAAATTTAGATGAATTGACTTT
PfAMA1.31	1	TTTGGTAAAGGTATAATTATTGAGAATTCAAATACTACTTTTTTAAAACCGGTAGCTACGGGAAATCAAGATTTAAAAGATGGAGGTTTTGCTTTTCCTCCAACAAATCCTCTTATATCACCAATGACATTAGATCATATGAGAGATTTTTATAAAAAAAATGAATATGTAAAAAATTTAGATGAATTGACTTT

PfAMA1.32	1	TTTGGTAAAGGTATAATTATTGAGAATTCAAATACTACTTTTTTAAAACCGGTAGCTACGGAAAATCAAGATTTAAAAGATGGAGGTTTTGCTTTTCCTCCAACAGAACCTCTTATATCACCAATGACATTAGATGATATGAGAGATTTTTATAAAAATAATGAATATGTAAAAAATTTAGATGAATTGACTTT
PfAMA1.33	1	TTTGGTAAAGGTATAATTATTGAGAATTCAAATACTACTTTTTTAAAACCGGTAGCTACGGGAAATCAACATTTAAAAGATGGAGGTTTTGCTTTTCCTCCAACAGAACCTCTTATATCACCAATGACATTAGATGATATGAGAGATTTTTATAAAAATAATGAATATGTAAAAAATTTAGATGAATTGACTTT
PfAMA1.34	1	TTTGGTAAAGGTATAATTATTGAGAATTCAAAAACTACTTTTTTAACACCGGTAGCTACGGAAAATCAAGATTTAAAAGATGGAGGTTTTGCTTTTCCTCCAACAAAACCTCTTATGTCACCAATGACATTAGATGATATGAGACTTTTGTATAAAGATAATGAATATGTAAAAAATTTAGATGAATTGACTTT

Supplemental Table 5. *sera2* Haplotypes Detected

Haplotype	# Samples Detected	Sequence
PfSERA2.00	29	TCCAGGTGATATCTTTGTTCCTACGGATCCTCCTGTTCCTGATGATCTTCCTGCTCTTTCGACATCTGATTGGGATACTTCTACACCTGATTTTGCTGATTCTACTTTTTGTTCTGCTCCTATACCACTTCCTACTTCTTTTTGTTGTTGCTGTTGTTGGGGTTGTGTTTCTTGAGCTAAAGTTGGTAATGCTGGTTGTTGTTTTGGTTTTTCAATTCTTGCTTGTGTTGTTACACGTGAACCATCGGATGATAATG
PfSERA2.01	22	TCCAGGTGATATCTTTGTTCCTACGGATCCTCCTGTTCCTGATGATCTTCCTGCTCTTTCGACATCTGATTGGGATACTTCTACACCTGGTTTTGCTGATTCTACTTTTTGTTCTGCTCCTATACCACTTCCTACTTCTTTTTGTTGTTGCTGTTGTTGGGGTTGTGTTTCTTGAGCTAAAGTTGGTAATGCTGGTTGTTGTTTTGGTTTTTCAATTCTTGCTTGTGTTGTTACACGTGAACCATCGGATGATAATG
PfSERA2.02	13	TCCAGGTGATATCTTTGTTCCTACGGATCCTCCTGTTCCTGATGATCTTCCTGCTCTTTCGACATCTGATTGGGATACTTCTGCACCTGGTTTTGCTGATTCTACTTTTTGTTCTGCTCCTATACCACTTCCTACTTCTTTTTGTTGTTGCTGTTGTTGGGGTTGTGTTTCTTGAGCTAAAGTTGGTAATGCTGGTTGTTGTTTTGGTTTTTCAATTCTTGCTTGTGTTGTTACACGTGAACCATCGGATGATAATG
PfSERA2.03	12	TCCAGGTGATATCTTTGTTCCTACGGATCCTCCTGTTCCTGATGATCTTCCTGCTCTTTCGACATCTGATTGGGATACTTCTGCACCTGGTCTTGCTGATTCTACTTTTTGTTCTGCTCCTATACCACTTCCTACTTCTTTTTGTTGTTGCTGTTGTTGGGGTTGTGTTTCTTGAGCTAAAGTTGGTAATGTTGGTTGTTGTTTTGGTTTTTCAATTCTTGCTTGTGTTGTTACACGTGAACCATCGGATGATAATG
PfSERA2.04	10	TCCAGGTGATATCTTTGTTCCTACGGATCCTCCTGTTCCTGATGATCTTCCTGCTCTTTCGACATCTGATTGGGATACTTCTACACCTGGTCTTGCTGATTCTACTTTTTGTTCTGCTCCTATACCACTTCCTACTTCTTTTTGTTGTTGCTGTTGTCGGGGTTGTGTTTCTTGAGCTAAAGTTGGTAATGCTGGTTGTTCTTTTGGTTTTTCAATTCTTGCTTGTGTTGTTACACGTGAACCATCGGATGATAATG
PfSERA2.05	9	TCCAGGTGATATCTTTGTTCCTACGGATCCTCCTGTTCCTGATGATCTTCCTGCTCTTTCGACATCTGATTGGGATACTTCTACACCTGGTTTTGCTAATTCTACTTTTTGTTCTGCTCCTATACCACTTCCTACTTCTTTTTGTTGTTGCTGTTGTTGGGGTTGTGTTTCTTGAGCTAAAGTTGGTAATGCTGGTTGTTGTTTTGGTTTTTCAATTCTTGCTTGTGTTGTTACACGTGAACCATCGGATGATAATG
PfSERA2.06	8	TCCAGGTGATATCTTTGTTCCTACGGATCCTCCTGTTCCTGATGATCTTCCTGCTCTTTCGACATCTGATTGGGATACTTCTACACCTGGTCTTGCTGATTCTACTTTTTGTTCTGCTCCTATACCACTTCCTACTTCTTTTTGTTGTTGCTGTTGTTGGGGTTGTGTTTCTTGAGCTAAAGTTGGTAATGCTGGTTGTTGTTTTGGTTTTTCAATTCTTGCTTGTGTTGTTACACGTGAACCATCGGATGATAATG
PfSERA2.07	7	TCCAGGTGATATCTTTGTTCCTACGGATCCTCCTGTTCCTGATGATCTTCCTGCTCTTTCGACATCTGATTGGGATACTTCTACACCTGATTTTGCTGATTCTACTTTTTGTTCTGCTCCTATACCACTTCCTACTTCTTTTTGTTGTTGCTGTTGTTGGGGTTGTGTTTCTTGAGCTAAAGTTGGTAATGCTGGTTGTTGTATTGGTTTTTCAATTCTTGCTTGTGTTGTTACACGTGAACCATCGGATGATAATG
PfSERA2.08	5	TCCAGGTGATATCTTTGTTCCTACGGATCCTCCTGTTCCTGATGATCTTCCTGCTCTTTCGACATCTGATTGGGATACTTCTACACCTGGTCTTGCTGATTCTACTTTTTGTTCTGCTCCTATACCACTTCCTACTTCTTTTTGTTGTTGCTGTTGTTGGGGTTGTGTTTCTTGAGCTAAAGTTGGTAATGCTGGTTGTTGTATTGGTTTTTCAATTCTTGCTTGTGTTGTTACACGTGAACCATCGGATGATAATG
PfSERA2.09	5	TCCAGGTGATATCTTTGTTCCTACGGATCCTCCTGTTCCTGATGATCTTCCTGCTCTTTCGACATCTGATTGGGATACTTCTACACCTGGTCTTGCTGATTCTACTTTTTGTTCTGCTCCTATACCACTTCCTACTTCTTTTTGTTGTTGCTGTTGTCGGGGTTGTGTTTCTTGAGCTAAAGTTGGTAATGCTGGTTGTTCTTTTGGTTCTTCAATTCTTGCTTGTGTTGTTACACGTGAACCATCGGATGATAATG
PfSERA2.10	4	TCCAGGTGATATCTTTGTTCCTACGGATCCTCCTGTTCCTGATGATCTTCCTGCTCTTTCGACATCTGATTGGGATACTTCTACACCTGGTATTGCTGATTCTACTTTTTGTTCTGCTCCTATACCACTTCCTACTTCTTTTTGTTGTTGCTGTTGTCGGGGTTGTGTTTCTTGAGCTAAAGTTGGTAATGCTGGTTGTTCTTTT

		GGTTTTTCAATTCTTGCTTGTGTTGTTACACGTGAACCATCGGATGATAATG
PfSERA2.11	4	TCCAGGTGATATCTTTGTTCCTACGGATCCTCCTGTTCCTGATGATCTTCCTGCTCTTTCGACATCTGATTGGGATACTTCTGCACCTGGTTTTGCTGATTC<br>TACTTTTTGTTCTGCTCCTATACCACTTCCTACTTCTTTTTGTTGTTGCTGTTGTTGGGGTTGTGTTTCTTGAGCTAAAGTTGGTAATGTTGGTTGTTGTTTT<br>GGTTTTTCAATTCTTGCTTGTGTTGTTACACGTGAACCATCGGATGATAATG
PfSERA2.12	3	TCCAGGTGATATCTTTGTTCCTACGGATCCTCCTGTTCCTGATGATCTTCCTGCTCTTTCGACATCTGATTGGGATACTTCTACACCTGGTTTTGCTGATTC<br>TACTTTTTGTTCTGCTCCTATACCACTTCCTACTTCTTTTTGTTGTTGCTGTTGTTGGGGTTGTGTTTCTTGAGCTAAAGTTGGTAATGCTCGTTGTTGTTTT<br>GGTTTTTCAATTCTTGCTTGTGTTGTTACACGTGAACCATCGGATGATAATG
PfSERA2.13	3	TCCAGGTGATATCTTTGTTCCTACGGATCCTCCTGTTCCTGATGATCTTCCTGCTCTTTCGACATCTGATTGGGATACTTCTACACCTGATTTTGCTGATTC<br>TACTTTTTGTTCTGCTCCTATACCACTTCCTACTTCTTTTTGTTGTTGCTGTTGTCGGGGTTGTGTTTCTTGAGCTAAAGTTGGTAATGCTGGTTGTTCTTTT<br>GGTTTTTCAATTCTTGCTTGTGTTGTTACACGTGAACCATCGGATGATAATG
PfSERA2.14	3	TCCAGGTGATATCTTTGTTCCTACGGATCCTCCTGTTCCTGATGATCTTCCTGCTCTTTCGACATCTGATTGGGATACTTCTACACCTGGTCTTGCTGATTC<br>TACTTTTTGTTCTGCTCCTATACCACTTCCTACTTCTTTTTGTTGTTGCTGTTGTCGGGGTTGTGTTTCTTGAGCTAAAGTTGGTAATGCTGATTGTTCTTTT<br>GGTTTTTCAATTCTTGCTTGTGTTGTTACACGTGAACCATCGGATGATAATG
PfSERA2.15	3	TCCAGGTGATATCTTTGTTCCTACGGATCCTCCTGTTCCTGATGATCTTCCTGCTCTTTCGACATCTGATTGGGATACTTCTACACCTGGTTTTGCTGATTC<br>TACTTTTTGTTCTGCTCCTATACCACTTCCTACTTCTTTTTGTTGTTGCTGTTGTTGGGGTTGTGTTTCTTGAGCTAAAGTTGGTAATGCTGGTTGTTGTTTT<br>TGTTTTTCAATTCTTGCTTGTGTTGTTACACGTGAACCATCGGATGATAATG
PfSERA2.16	3	TCCAGGTGATATCTTTGTTCCTACGGATCCTCCTGTTCCTGATGATCTTCCTGCTCTTTCGACATCTGATTGGGATACTTCTGCACCTGGTTTTGCTGATTC<br>TACTTTTTGTTCTGCTCCTATACCACTTCCTACTTCTTTTTGTTGTTGCTGTTGTTGGGGTTGTGTTTCTTGAGCTAAAGTTGGTAATGCTGGTTGTTGTATT<br>GGTTTTTCAATTCTTGCTTGTGTTGTTACACGTGAACCATCGGATGATAATG
PfSERA2.17	2	TCCAGGTGATATCTTTGTTCCTACGGATCCTCCTGTTCCTGATGATCTTCCTGCTCTTTCGACATCTGATTGGGATACTTCTGCACCTGGTCTTGCTGATTC<br>TACTTTTTGTTCTGCTCCTATACCACTTCCTACTTCTTTTTGTTGTTGCTGTTGTCGGGGTTGTGTTTCTTGAGCTAAAGTTGGTAATGCTGGTTGTTCTTTT<br>GGTTTTTCAATTCTTGCTTGTGTTGTTACACGTGAACCATCGGATGATAATG
PfSERA2.18	2	TCCAGGTGATATCTTTGTTCCTACGGATCCTCCTGTTCCTGATGATCTTCCTGCTCTTTCGACATCTGATTGGGATACTTCTGCATCTGGTTTTGCTGATTC<br>TACTTTTTGTTCTGCTCCTATACCACTTCCTACTTCTTTTTGTTGTTGCTGTTGTTGGGGTTGTGTTTCTTGAGCTAAAGTTGGTAATGCTGGTTGGTCTTT<br>TGGTTTTTCAATTCTTGCTTGTGTTGTTACACGTGAACCATCGGATGATAATG
PfSERA2.19	2	TCCAGGTGATATCTTTGTTCCTACGGATCCTCCTGTTCCTGATGATCTTCCTGCTCTTTCGACATCTGATTGGGATACTTCTACACCTGGTTTTGCTGATTC<br>TACTTTTTGTTCTGCTCCTATACCACTTCCTACTTCTTTTTGTTGTTGCTGTTGTTGGGGTTGTGTTTCTTGAGCTAAAGTTGGTAATTCTGGTTGTTGTTTT<br>GGTTTTTCAATTCTTGCTTGTGTTGTTACACGTGAACCATCGGATGATAATG
PfSERA2.20	2	TCCAGGTGATATCTTTGTTCCTACGGATCCTCCTGTTCCTGATGATCTTCCTGCTCTTTCGACATCTGATTGGGATACTTCTACACCTGGTTTTGCTAATTC<br>TACTTTTTGTTCTGCTCCTATACCACTTCCTACTTCTTTTTGTTGTTGCTGTTGTCGGGGTTGTGTTTCTTGAGCTAAAGTTGGTAATGCTGGTTGTTCTTTT<br>GGTTTTTCAATTCTTGCTTGTGTTGTTACACGTGAACCATCGGATGATAATG
PfSERA2.21	2	TCCAGGTGATATCTTTGTTCCTACGGATCCTCCTGTTCCTGATGATCTTCCTGCTCTTTCGACATCTGATTGGGATACTTCTGCACCTGGTTTTGCTGATTC<br>TACTTTTTGTTCTGCTCCTATACCACTTCCTACTTCTTTTTGTTGTTGCTGTTGTTGGGGTTGTGTTTCTTGAGCTAAAGTTGGTAATGCTGGTTGTTCTTTT<br>GGTTTTTCAATTCTTGCTTGTGTTGTTACACGTGAACCATCGGATGATAATG
PfSERA2.22	1	TCCAGGTGATATCTTTGTTCCTACGGATCCTCCTGTTCCTGATGATCTTCCTGCTCTTTCGACATTTGATTGGGATACTTCTACACCTGGTCTTGCTGATTC<br>TACTTTTTGTTCTGCTCCTATACCACTTCCTACTTCTTTTTGTTGTTGCTGTTGTCGGGGTTGTGTTTCTTGAGCTAAAGTTGGTAATGCTGGTTGTTCTTTT

		GGTTCTTCAATTCTTGCTTGTGTTGTTACACGTGAACCATCGGATGATAATG
PfSERA2.23	1	TCCAGGTGATATCTTTGTTCCTACGGATCCTCCTGTTCCTGATGATCTTCCTGCTCTTTCGACATCTGATTGGGATACTTCTACACCTGGTTTTGCTAATTC<br>TACTTTTTGTTCTGCTCCTATACCACTTCCTACTTCTTTTTGTTGTTGCTGTTGTCGGGGTTGTGTTTCTTGAGCTAAAGTTGGTAATGCTGATTGTTCTTTT<br>GGTTTTTCAATTCTTGCTTGTGTTGTTACACGTGAACCATCGGATGATAATG
PfSERA2.24	1	TCCAGGTGATATCTTTGTTCCTACGGATCCTCCTGTTCCTGATGATCTTCCTGCTCTTTCGACATCTGATTGGGATACTTCTACACCTGGTCTTGCTGATTC<br>TACTTTTTGTTCTGCTCCTATACCACTTCCTACTTCTTTTTGTTGTTGCTGTTGTTGGGGTTGTGTTTCTTGAGCTAAAGTTGGTAATTCTGGTTGTTGTTTT<br>GGTTTTTCAATTCTTGCTTGTGTTGTTACACGTGAACCATCGGATGATAATG
PfSERA2.25	1	TCCAGGTGATATCTTTGTTCCTACGGATCCTCCTGTTCCTGATGATCTTCCTGCTCTTTCGACATCTGATTGGGATCCTTCTACACCTGGTTTTGCTGATTC<br>TACTTTTTGTTCTGCTCCTATACCACTTCCTACTTCTTTTTGTTGTTGCTGTTGTTGGGGTTGTGTTTCTTGAGCTAAAGTTGGTAATGCTGGTTGTTGTTTT<br>GGTTTTTCAATTCTTGCTTGTGTTGTTACACGTGAACCATCGGATGATAATG
PfSERA2.26	1	TCCAGGTGATATCTTTGTTCCTACGGATCCTCCTGTTCCTGATGATCTTCCTGCTCTTTCGACATCTGATTGGGATACTTCTACACCTGGTATTGCTGATTC<br>TACTTTTTGTTCTGCTCCTATACCACTTCCTACTTCTTTTTGTTGTTGCTGTTGTTGGGGTTGTGTTTCTTGAGCTAAAGTTGGTAATGCTGGTTGTTGTTTT<br>TGTTTTTCAATTCTTGCTTGTGTTGTTACACGTGAACCATCGGATGATAATG
PfSERA2.27	1	TCCAGGTGATATCTTTGTTCCTACGGATCCTCCTGTTCCTGATGATCTTCCTGCTCTTTCGACATCTGATTGGGATACTTCTACACCTGATTTTGCTGATTC<br>TACTTTTTGTTCTGCTCCTATACCACTTCCTACTTCTTTTTGTTGTTGCTGTTGTCGGGGTTGTGTTTCTTGAGCTAAAGTTGGTAATGCTGGTTCTTTTGG<br>TTTTTCAATTCTTGCTTGTGTTGTTACACGTGAACCATCGGATGATAATG
PfSERA2.28	1	TCCAGGTGATATCTTTGTTCCTACGGATCCTCCTGTTCCTGATGATCTTCCTGCTCTTTCGACATCTGATTTGGATACTTCTACACCTGATTTTGCTGATTCT<br>ACTTTTTGTTCTGCTCCTATACCACTTCCTACTTCTTTTTGTTGTTGCTGTTGTTGGGGTTGTGTTTCTTGAGCTAAAGTTGGTAATGCTGGTTGTTGTTTT<br>GGTTTTTCAATTCTTGCTTGTGTTGTTACACGTGAACCATCGGATGATAATG

Supplemental Table 6. *trap* Haplotypes Detected

Haplotype	# Samples Detected	Sequence
PfTRAP.00	3	TAATCCTCCAGCTATTCCACCTGCAATTTTATATTTATTATCTGATCCTCCTTTTTGTTTATTATTATCTGGCTTTTCATGTTCTTCCCTTTCAGGATGTTTT<br>GGAGTATTGTTATGTTTTCTATTGTATGATCTATTTTCATTATTTCTACCATGTGGACGTGTTTCTCTATCTTCACTATTAGGTACGTGCCTATTTCCATTAT<br>TATCTTGACTTTGGGGGTCACTTTGTTTCCTTTCATTATCCAAAACTTTTGGAGGTAATGGTGAATATGGAATATTTCTATCACTTTTATCATTTGGTAAAT<br>TATTTTGATT
PfTRAP.01	2	TAATCCTCCAGCTATTCCACCTGCAATTTTATATTTATTATCTGATCCTGCTTTTTTTTTATTATTATCTGGCTTTTCATGTTCTTCCCTTTCAGGATGTTTT<br>GGAGTATCGTTATGTTTTCTATTGTATGATCTATTTTCATTATTTCTACCATGTGGACGTGTTTCTCTATCTTCACTATTAGGTACGTGCCTATTTCCATTAT<br>TATCTTGACTTTGGGGGTCACTTTGTTTCCTTTCATTATCCAAAACATTTGGAGGTAATGGTGAATATGGAATATATCTATCACTTTTATCATTTGGTAAAT<br>TATTTTGATT
PfTRAP.02	1	TAATCCTCCAGCTATTCCACCTGCAATTTTATATTTATTATCTGATCCTCCTTTTTTTTTATTATTATCTGGCTTTTCATGTTCTTCCCTTTCAGGATATTTT<br>GGAGTATTGTTATGTTTTCTATTGTATGATCTATTTTCATTATTTCTACCATGTGGACGTGTTTCTCTATCTTCACTATTAGGTACGTGCCTATTTCCATTAT<br>TATCTTGACTTTGGGGGTCACTTTGTTTCCTTTCATTATCCAAAACATTTGGAGGTAATGGTGAATATGGAATATATCTATCACTTTTATTATTTGGTAAAT<br>TATTTTGATT
PfTRAP.03	1	TAATCCTCCAGCTATTCCACCTGCAATTTTATATTTATTATCTGATCCTCCTTTTTTTTTATTATTATCTGGCTTTTCATGTTCTTCCCTTTCAGGATGTTTT<br>GGAGTATCGTTATGTTTTCTATTGTATGATCTATTTTCATTATTTCTACCATGTGGACGTGTTTCTCTATCTTCACTATTAGGTACGTGCCTATTTCCATTAT<br>TATCTTGACTTTGGGGGTCACTTTGTTTCCTTTCATTATCCAAAACATTTGGAGGTAATGGTGAATATGGAATATATCTATCACTTTTATCATTTGGTAAAT<br>TATTTTGATT
PfTRAP.04	1	TAATCCTCCAGCTATTCCACCTGCAATTTTATATTTATTATCTGATCCTCCTTTTTGTTTATTATTATCTGGCTTTTCATGTTCTTCCCTTTCATGATATTTTG<br>GAGTATCGTTATGTTTTCTATTGTATGATCTATTTTCATTATTTCTACCATGTGGATGTGTTTCTCTATCTTCACTATTAGGTACGTGCCTATTTCCATTATT<br>ATCTTGACTTTGGGGGTCACTTTGTTTCCTTTCATTATCCAAAACTTTTGGAGGTAATGGTGAATATGGAATATATCTATCACTTTTATCATTTGGTAAATT<br>ATTTTGATT
PfTRAP.05	1	TAATCCTCCAGCTATTCCACCTGCAATTTTATATTTATTATCTGATCCTCCTTTTTGTTTATTATTATCTGGCTTTTCATGTTCTTCCCTTTCAGGATGTTTT<br>GGAGTATTGTTATGTTTTCTATTGTATGATCTATTTTCATTATTTCTACCATGTGGACGTGTTTCTCTATCTTCACTATTAGGTACGTGCCTATTTCCATTAT<br>TATCTTGACTTTGGGGGTCACTTTGTTTCCTTTCATTATCCAAAACATTTGGAGGTAATGGTGAATATGGAATATATCTATCACTTTTATCATTTGGTAAAT<br>TATTTTGATT
PfTRAP.06	1	TAATCCTCCAGCTATTCCACCTGCAATTTTATATTTATTATCTGATGCTCCTTTTTTTTTATTATTATCTGGCTTTTCATGTTCTTCCCTTTCAGGATATTTT<br>GGAGTATCGTTATATTTTATATTGTATGATCTATTTTCATTATTTCTACCATGTGGACGTGTTTCTCTATCTTCACTATTAGGTACGTGCCTATTTCCATTAT<br>TATCTTGACTTTGGGGGTCACTTTGTTTCCTTTCATTATCCAAAACTTTTGGAGGTAATGGTGAATATGGAATATATCTATCACTTTTATCATTTGGTAAAT<br>TATTTTGATT
PfTRAP.07	1	TAATCCTCCAGCTATTCCACCTGCAATTTTGTATTTATTATCTGATCCTCCTTTTTGTTTATTATTATCTGGCTTTTCATGTTCTTCCCTTTCAGGATGTTTT<br>GGAGTATTGTTATGTTTTCTATTGTATGATCTATTTTCATTATTTCTACCATGTGGACGTGTTTCTCTATCTTCACTATTAGGTACGTGCCTATTTCCATTAT<br>TATCTTGACTTTGGGGGTCACTTTGTTTCCTTTCATTATCCAAAACTTTTGGAGGTAATGGTGAATATGGAATATATCTATCACTTTTATCATTTGGTAAAT<br>TATTTTGATT
PfTRAP.08	1	TAATCCTCCAGCTATTCCACCTGCAATTTTATATTTATTATCTGATCCTCCTTTTTTTTTATTATTATCTGGCTTTTCATGTTCTTCCCTTTCATGATATTTTG

		GAGTATCGTTATGTTTTCTATTGTATGATCTATTTTCATTATTTCTACCATGTGGATGTGTTTCTCTATCTTCACTATTAGGTACGTGCCTATTTCCATTATT ATCTTGACTTTGGGGGTCACTTTGTTTCCTTTCATTATCCAAAACTTTTGGAGGTAATGGTGAATATGGAATATATCTATCACTTTTATCATTTGGTAAATT ATTTTGATT
PfTRAP.09	1	TAATCCTCCAGCTATTCCACCTGCAATTTTATATTTATTATCTGATCCTCCTTTTTGTTTATTATTATCTGGCTTTTCATGTTCTTCCCTTTCATGATATTTTG GAGTATTGTTATGTTTTCTATTGTATGATCTATTTTCATTATTTCTACCATGTGGACGTGTTTCTCTATCTTCACTATTAGGTACGTGCCTATTTCCATTATT ATCTTGACTTTGGGGGTCACTTTGTTTCCTTTCATTATCCAAAACATTTGGAGGTAATGGTGAATATGGAATATATCTATCACTTTTATCATTTGGTAAATT ATTTTGATT

Supplemental Table 7. Heterozygosity Contributions. For each gene, the table shows each haplotype, the number of samples detected, its frequency in the population, and its squared frequency (contribution to homozygosity).

csp				***ama1***				***sera2***				***trap***			
Haplotype	# Samples Detected	Frequency	Contribution_to_Homozygosity	Haplotype	# Samples Detected	Frequency	Contribution_to_Homozygosity	Haplotype	# Samples Detected	Frequency	Contribution_to_Homozygosity	Haplotype	# Samples Detected	Frequency	Contribution_to_Homozygosity
PfCSP.00	9	2.00E-01	4.00E-02	PfAMA1.00	23	9.75E-02	9.50E-03	PfSERA2.00	29	1.81E-01	3.29E-02	PfTRAP.00	3	2.31E-01	5.33E-02
PfCSP.01	7	1.56E-01	2.42E-02	PfAMA1.01	18	7.63E-02	5.82E-03	PfSERA2.01	22	1.38E-01	1.89E-02	PfTRAP.01	2	1.54E-01	2.37E-02
PfCSP.02	4	8.89E-02	7.90E-03	PfAMA1.02	17	7.20E-02	5.19E-03	PfSERA2.02	13	8.13E-02	6.60E-03	PfTRAP.02	1	7.69E-02	5.92E-03
PfCSP.03	3	6.67E-02	4.44E-03	PfAMA1.03	16	6.78E-02	4.60E-03	PfSERA2.03	12	7.50E-02	5.63E-03	PfTRAP.03	1	7.69E-02	5.92E-03
PfCSP.04	3	6.67E-02	4.44E-03	PfAMA1.04	15	6.36E-02	4.04E-03	PfSERA2.04	10	6.25E-02	3.91E-03	PfTRAP.04	1	7.69E-02	5.92E-03
PfCSP.05	2	4.44E-02	1.98E-03	PfAMA1.05	12	5.08E-02	2.59E-03	PfSERA2.05	9	5.63E-02	3.16E-03	PfTRAP.05	1	7.69E-02	5.92E-03
PfCSP.06	2	4.44E-02	1.98E-03	PfAMA1.06	11	4.66E-02	2.17E-03	PfSERA2.06	8	5.00E-02	2.50E-03	PfTRAP.06	1	7.69E-02	5.92E-03
PfCSP.07	1	2.22E-02	4.94E-04	PfAMA1.07	11	4.66E-02	2.17E-03	PfSERA2.07	7	4.38E-02	1.91E-03	PfTRAP.07	1	7.69E-02	5.92E-03
PfCSP.08	1	2.22E-02	4.94E-04	PfAMA1.08	11	4.66E-02	2.17E-03	PfSERA2.08	5	3.13E-02	9.77E-04	PfTRAP.08	1	7.69E-02	5.92E-03
PfCSP.09	1	2.22E-02	4.94E-04	PfAMA1.09	10	4.24E-02	1.80E-03	PfSERA2.09	5	3.13E-02	9.77E-04	PfTRAP.09	1	7.69E-02	5.92E-03
PfCSP.10	1	2.22E-02	4.94E-04	PfAMA1.10	9	3.81E-02	1.45E-03	PfSERA2.10	4	2.50E-02	6.25E-04				
PfCSP.11	1	2.22E-02	4.94E-04	PfAMA1.11	9	3.81E-02	1.45E-03	PfSERA2.11	4	2.50E-02	6.25E-04				
PfCSP.12	1	2.22E-02	4.94E-04	PfAMA1.12	6	2.54E-02	6.46E-04	PfSERA2.12	3	1.88E-02	3.52E-04				
PfCSP.13	1	2.22E-02	4.94E-04	PfAMA1.13	6	2.54E-02	6.46E-04	PfSERA2.13	3	1.88E-02	3.52E-04				

PfCSP. 14	1	2.22E-02	4.94E-04	PfAMA 1.14	6	2.54E-02	6.46E-04	PfSERA 2.14	3	1.88E-02	3.52E-04				
PfCSP. 15	1	2.22E-02	4.94E-04	PfAMA 1.15	5	2.12E-02	4.49E-04	PfSERA 2.15	3	1.88E-02	3.52E-04				
PfCSP. 16	1	2.22E-02	4.94E-04	PfAMA 1.16	5	2.12E-02	4.49E-04	PfSERA 2.16	3	1.88E-02	3.52E-04				
PfCSP. 17	1	2.22E-02	4.94E-04	PfAMA 1.17	5	2.12E-02	4.49E-04	PfSERA 2.17	2	1.25E-02	1.56E-04				
PfCSP. 18	1	2.22E-02	4.94E-04	PfAMA 1.18	4	1.69E-02	2.87E-04	PfSERA 2.18	2	1.25E-02	1.56E-04				
PfCSP. 19	1	2.22E-02	4.94E-04	PfAMA 1.19	4	1.69E-02	2.87E-04	PfSERA 2.19	2	1.25E-02	1.56E-04				
PfCSP. 20	1	2.22E-02	4.94E-04	PfAMA 1.20	4	1.69E-02	2.87E-04	PfSERA 2.20	2	1.25E-02	1.56E-04				
PfCSP. 21	1	2.22E-02	4.94E-04	PfAMA 1.21	4	1.69E-02	2.87E-04	PfSERA 2.21	2	1.25E-02	1.56E-04				
				PfAMA 1.22	3	1.27E-02	1.62E-04	PfSERA 2.22	1	6.25E-03	3.91E-05				
				PfAMA 1.23	3	1.27E-02	1.62E-04	PfSERA 2.23	1	6.25E-03	3.91E-05				
				PfAMA 1.24	3	1.27E-02	1.62E-04	PfSERA 2.24	1	6.25E-03	3.91E-05				
				PfAMA 1.25	2	8.47E-03	7.18E-05	PfSERA 2.25	1	6.25E-03	3.91E-05				
				PfAMA 1.26	2	8.47E-03	7.18E-05	PfSERA 2.26	1	6.25E-03	3.91E-05				
				PfAMA 1.27	2	8.47E-03	7.18E-05	PfSERA 2.27	1	6.25E-03	3.91E-05				
				PfAMA 1.28	2	8.47E-03	7.18E-05	PfSERA 2.28	1	6.25E-03	3.91E-05				
				PfAMA 1.29	2	8.47E-03	7.18E-05								
				PfAMA 1.30	2	8.47E-03	7.18E-05								

				PfAMA 1.31	1	4.24E-03	1.80E-05								
				PfAMA 1.32	1	4.24E-03	1.80E-05								
				PfAMA 1.33	1	4.24E-03	1.80E-05								
				PfAMA 1.34	1	4.24E-03	1.80E-05								

Supplemental Table 8. Public *csp* sequences used in analysis

Study	Genbank Identifier
Foko, LPK, et al. https://doi.org/10.1016/j.gene.2024.148744	OQ709066.1
	OQ709065.1
	OQ709064.1
	OQ709063.1
	OQ709062.1
	OQ709061.1
	OQ709060.1
	OQ709059.1
	OQ709058.1
	OQ709057.1
	OQ709056.1
	OQ709055.1
	OQ709054.1
	OQ709053.1
	OQ709052.1
	OQ709051.1
	OQ709050.1
	OQ709049.1
	OQ709048.1
	OQ709047.1
	OQ709046.1
	OQ709045.1

Supplemental Figure 1. Protein Alignment of CS Haplotypes. Numbers correspond to nucleotide haplotype number.

```
20      DANANANNAVKNNNNEEPSDQHIEKYIKKIQNSLSTEWSPCSVTCGNGIQVRIKPGSADK
1       DENANANSAVKNNNNEEPSDKHIKEYLNKIQNSLSTEWSPCSVTCGNGIQVRIKPGSANK
21      DENANANSAVKNNNNEEPSDKHIKEYLNKIQNSISTEWSPCSVTCGNGIQVRIKPGSANK
5       DENANANNAVKNNNNEEPSDKHIEQYLKKIQNSLSTEWSPCSVTCGNGIQVRIKPGSAGK
19      DENANANNAVKNNNNEEPSDKHIEQYLKTIKNSLSTEWSPCSVTCGNGIQVRIKPGSANK
10      DENAKANNAVKNNNNEEPSDKHIEQYLKTIKNSLSTEWSPCSVTCGNGIQVRIKPGSANK
0       DENAKANNAVKNNNNEEPSDKHIEQYLKTIQNSLSTEWSPCSVTCGNGIQVRIKPGSANK
16      DENAKANNAVKNNNNEEPSDKHIEQYLKTIQNSLSTEWSPCSVTCGNGIQVRIKPGSANK
18      DENANANNAVKNNNNEEPSDQHIEKYLKRIQNSLSTEWSPCSVTCGNGIQVRIKPGSANK
14      DENANGNNAVKNNNNEEPSDQHIEKYLKKIQNSLSTEWSPCSVTCGNGIQVRIKPGSADK
2       DENANANNAVKNNNNEEPSDQHIEKYLNKIKNSLSTEWSPCSVTCGNGIQVRIKPGSANK
11      DENANANNAVKNNNNEEPSDQHIEKYLKRIQNSLSTEWSPCSVTCGNGIQVRIKPGSADK
12      DENANANNAVKNNNNEEPSDQHIEKYLKRIQNSLSTEWSPCSVTCGNGIQVRIKPGSANK
17      DENAKANNAVKNNNNEEPSDQHIEKYLKTIQNSLSTEWSPCSVTCGNGIQVRIKPGSANK
3       DENANANNAVKNNNNEEPSDQHIEKYLKTIQNSLSTEWSPCSVTCGNGIQVRIKPGSANK
6       DENANANNAVKNNNNEEPSDQHIEKYLKIIKNSLSTEWSPCSVTCGNGIQVRIKPGSANK
4       DENANANNAVKNNNNEEPSDQHIEKYLKTIKNSLSTEWSPCSVTCGNGIQVRIKPGSANK
15      DENANANNAVKNNNNEEPSDKHIKEYLKRIQNSISTEWSPCSVTCGNGIQVRIKPGSAGK
7       DENANANNAVKNNNNEEPSDKHIEQYLNKIKNSISTEWSPCSVTCGNGIQVRIKPGSANK
13      DENANANNAVKNNNNEEPSDKHIEEYLKKIKNSISTEWSPCSVTCGNGIQVRIKPGSANK
8       DENANSNNAVKNNNNEEPSDKHITEYLKRIQNSLSTEWSPCSVTCGNGIQVRIKPGSANK
9       DENANGNNAVKNNNNEEPSDKHITEYLKRIQNSLSTEWSPCSVTCGNGIQVRIKPGSAGK
        * **:.*.*************:** :*:: *:**:*************************.*

20      PKNELDYENDIEKKICKMEKCSSVFNVVNSSIGLI
1       PKDELDYANDIEKKICKMEKCSSVFNVVNSSIGLI
21      PKDQLDYANDIEKKICKMEKCSSVFNVVNSSIGLI
5       SKDELDYENDIEKKICKMEKCSSVFNVVNSSIGLI
19      PKDQLDYENDIEKKICKMEKCSSVFNVVNSSIGLI
10      PKDQLDYENDIEKKICKMEKCSSVFNVVNSSIGLI
0       PKDQLDYENDIEKKICKMEKCSSVFNVVNSSIGLI
16      PKDELDYENDIEKKICKMEKCSSVFNVVNSSIGLI
18      PKDELNYENDIEKKICKMEKCSSVFNVVNSSIGLI
14      PKDQLDYANDIEKKICKMEKCSSVFNVVNSSIGLI
2       PKDQLDYANDIEKKICKMEKCSSVFNVVNSSIGLI
11      PKDQLDYANDIEKKICKMEKCSSVFNVVNSSIGLI
12      PKDQLDYANDIEKKICKMEKCSSVFNVVNSSIGLI
17      PKDQLDYANDIEKKICKMEKCSSVFNVVNSSIGLI
3       PKDQLDYANDIEKKICKMEKCSSVFNVVNSSIGLI
6       PKDQLDYANDIEKKICKMEKCSSVFNVVNSSIGLI
4       PKDQLDYANDIEKKICKMEKCSSVFNVVNSSIGLI
15      PKDELNYENDIEKKICKMEKCSSVFNVVNSSIGLI
7       PKDELNYENDIEKKICKMEKCSSVFNVVNSSIGLI
13      PKDELNYENDIEKKICKMEKCSSVFNVVNSSIGLI
8       PKEELNYENDIEKKICKMEKCSSVFNVVNSSIGLI
9       SKNELDYENDIEKKICKMEKCSSVFNVVNSSIGLI
        .*::*:* ****************************
```

Supplemental Figure 2. Within-Participant Nucleotide Haplotype Diversity. The number of haplotypes detected (y-axis) for each amplicon is shown for all 100 samples (x-axis).

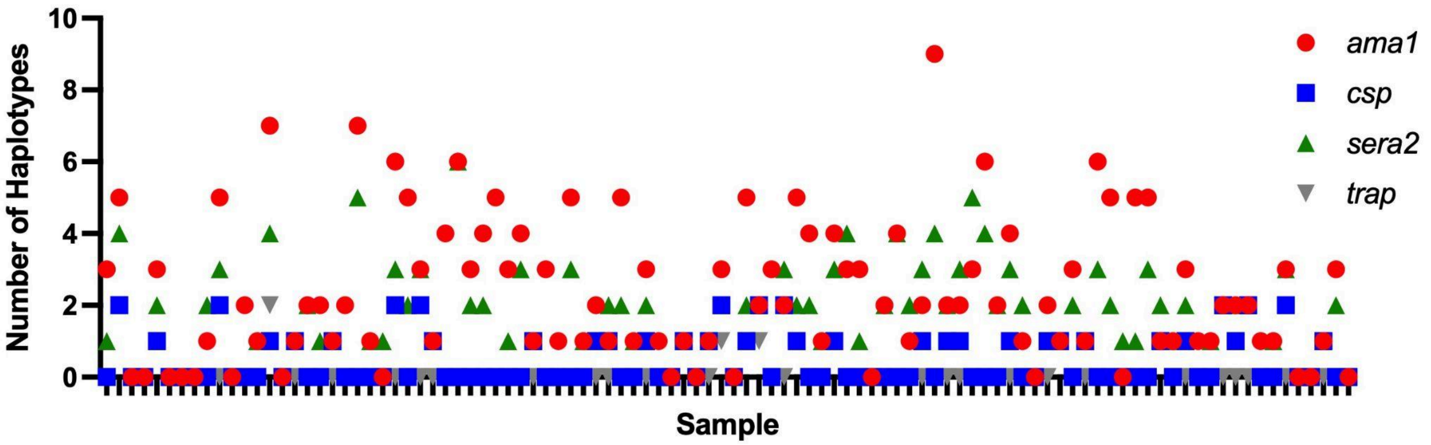

Supplemental Figure 3. Amino Acid Frequency in TH2 and TH3 Epitopes. Frequency determined by presence in invection, not accounting for within-sample allele frequency.

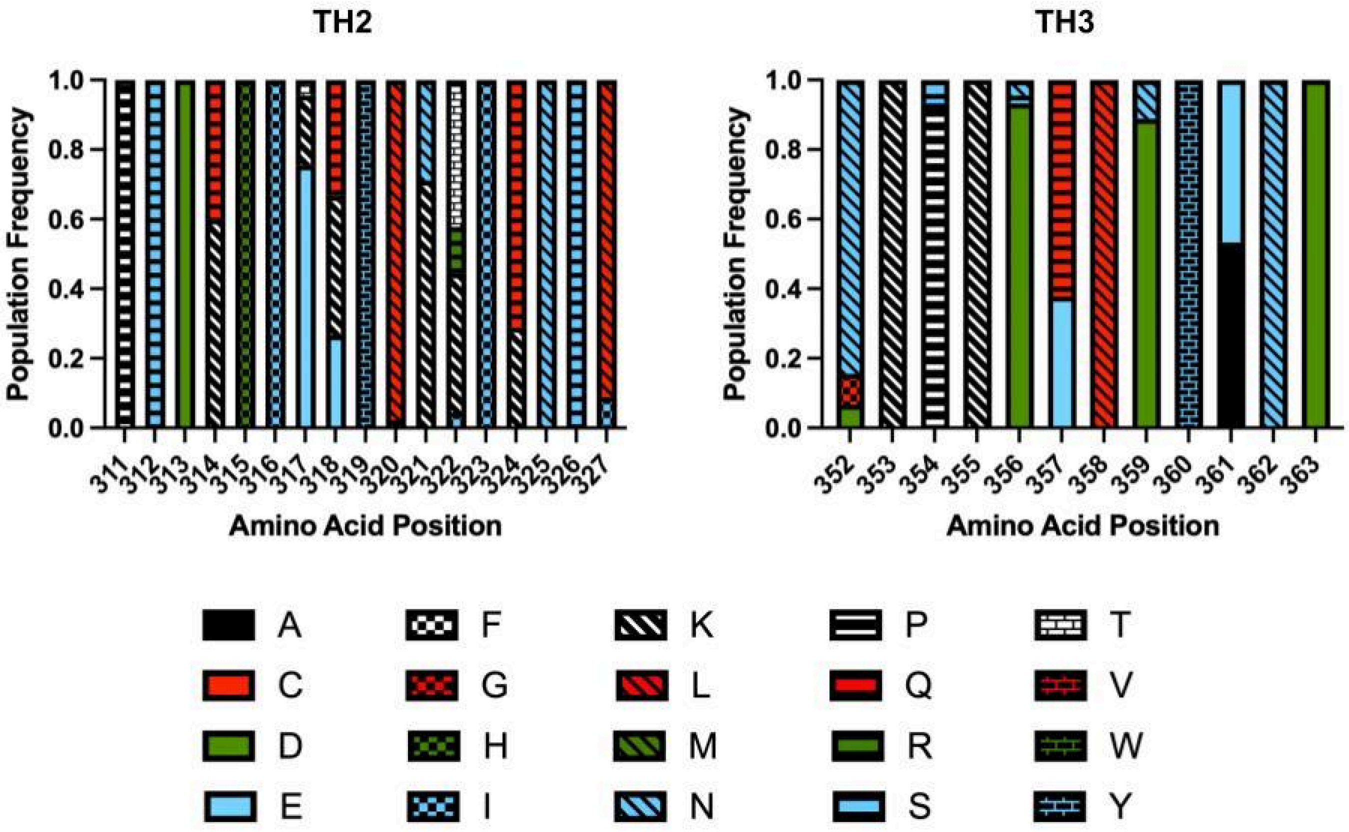

Supplemental Figure 2. Sliding Window of Tajima's D for *ama1* (Panel A), *sera2* (Panel B), and *trap* (Panel C). Data generated with a window size of 25bp and a slide of 5bp.Significant windows are marked with an *.

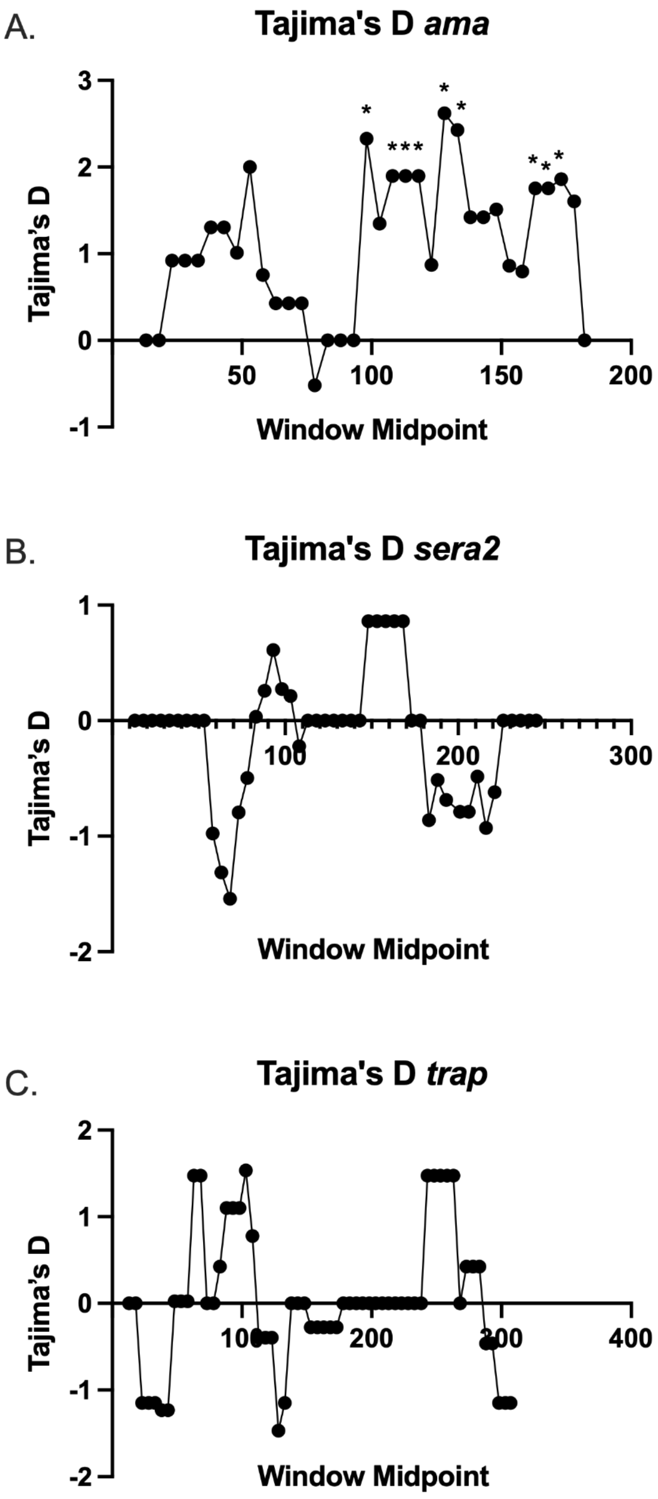

Supplement: Supplementary file 1 [file DataSheet1.pdf]
